# Efficacy of telemedicine on glycaemic control in nursing home residents with type 2 diabetes on basal‐bolus insulin therapy: A randomised controlled trial

**DOI:** 10.1111/dom.70511

**Published:** 2026-01-26

**Authors:** Ilaria Dicembrini, Chiara D. Poggi, Gloria G. Del Vescovo, Christian Marinelli, Daniele Scoccimarro, Valentina Vitale, Giovanni A. Silverii, Luca Drigani, Francesca Pancani, Roberto Norgiolini, Graziano Di Cianni, Edoardo Mannucci

**Affiliations:** ^1^ Experimental and Clinical Biomedical Sciences ‘Mario Serio’ University of Florence Florence Italy; ^2^ Diabetes Department, Local Health Unit North‐West Tuscany Livorno Hospital Livorno Italy; ^3^ Diabetes Unit USL Umbria 1 Perugia Italy

**Keywords:** continuous glucose monitoring (CGM), glycaemic control, hypoglycaemia, insulin treatment, telemedicine

## Abstract

**Aims:**

Management of insulin therapy in elderly individuals with type 2 diabetes (T2D) residing in nursing homes is often challenging due to comorbidities, cognitive impairment and limited access to specialist care. Continuous glucose monitoring (CGM) and telemedicine may help optimise glycaemic control in this vulnerable population.

**Materials and Methods:**

In order to assess the efficacy and safety of a CGM and telemedicine‐based management of insulin therapy in nursing home residents with T2D, a 12‐week, randomised, controlled and open‐label trial has been designed. Eighty‐five patients on stable basal‐bolus insulin therapy were assigned to either telemedicine‐assisted insulin titration based on CGM data (intervention group) or standard care with capillary blood glucose monitoring (control group). The primary endpoint was the change in time in range (TIR, 70–180 mg/dL), with secondary outcomes including time below range (TBR), time above range (TAR), haemoglobin A1c (HbA1c), insulin dose and safety endpoints.

**Results:**

TIR increased significantly in the intervention, but not in the control group, with a significant difference between study groups (*p* = 0.010). TBR showed a reduction in the intervention arm and an increase in the control arm with a significant difference between groups (*p* = 0.007). HbA1c and mean insulin daily units significantly also decreased in the intervention group, with significant differences between groups (*p* = 0.028 and *p* = 0.002, respectively). No safety issues potentially related to the intervention were identified during the study.

**Conclusion:**

In conclusion, remote insulin dose adjustment based on interstitial glucose monitoring ameliorates glucose control in nursing home residents with T2D on basal‐bolus insulin therapy.

## 1 INTRODUCTION

Despite the availability of many alternative therapeutic options, a relevant fraction of older people with type 2 diabetes (T2D) needs a basal‐bolus insulin treatment.^1^ The management of multiple daily injections of insulin is challenging and it includes relatively frequent insulin dose titration.^2^ Nursing home residents represent a population at risk for mismanagement because of multiple comorbidities (including cognitive dysfunction, functional disability and irregular nutritional intake) and greater difficulty in accessing office visits and specialist care.^3^

The availability of simple and inexpensive devices for continuous monitoring of interstitial glucose (continuous glucose monitoring [CGM]) is modifying the usual management of insulin‐treated diabetes. Such systems allow for greater accuracy in the adjustment of insulin doses, leading to an improvement of glycaemic control and reducing both hyper‐ and hypoglycaemia. These effects have been widely demonstrated in people with type 1 diabetes^4^; however, a growing body of evidence suggests possible clinical advantages for CGM also in some categories of patients with T2D.^5^ CGM systems usually allow for the upload of glucose data on remote servers, which can be used for transmission to health professionals, offering the opportunity of developing telemedicine systems. Such communication, if associated with a remote adjustment of insulin doses based on automatically transmitted sensor data, when applied to nursing home residents, could overcome the obstacles to access specialist office visits.

The present study is aimed at the assessment of the efficacy and safety of telemedicine‐based management of basal‐bolus insulin therapy in nursing home residents, using the possibilities offered by a CGM system.

## 2 MATERIALS AND METHODS

This trial used a randomised, controlled, open‐label design, with a 12‐week study period.

The trial is reported following CONSORT guideline^6^ (Figure S1).

Adults with T2D were eligible if they were older than 65 years, resident in nursing homes (National Health System‐affiliated long‐term care facilities, listed in Figure S2), and treated with a basal‐bolus insulin regimen for at least 3 months. The protocol was approved by the local Ethical Board Committee (19587‐PF) and was never amended. Informed consent was obtained from participants prior to enrolment. Consents from participants with cognitive impairment were collected from a surrogate decision‐maker.

Investigators randomised participants 1:1 to intervention or control arms, using a computer‐based randomisation sequence. Patients in the intervention group performed CGM monitoring (Freestyle Libre©, Abbott) during the entire study period; adjustment of insulin doses was performed remotely by a specialist (ID, CDP), using information from CGM profiles on a weekly basis.

For CGM data transmission, the LibreView© Digital Software application was installed at each nursing home. No pre‐defined algorithm was used for insulin dose titration, leaving clinical decisions to the responsibility and judgment of each investigator.

Patients assigned to the control arm (Standard of Care [SoC]) performed their usual capillary blood glucose monitoring; insulin doses were adjusted, if needed, by their healthcare providers (i.e., general practitioners in charge), as for SoC. The physicians responsible for diabetes care in these patients were their general practitioners, who had free access to remote consultancies with diabetes specialists and the possibility to prescribe specialist diabetes visits free of charge for their patients whenever deemed necessary. Patients in the control arm wore a blinded CGM device (Freestyle Libre Pro©, Abbott) for 14 days at the beginning of the study and at Week 10; data from those sensors, which were not available to patients or to their healthcare providers, were downloaded by the investigators only at the end of the study.

Patients, nurses and investigators were all aware of treatment arm assignment, since blinding was not possible.

The research team performed a 2‐h educational meeting for the nursing staff at the long‐term care facilities involved. The nursing staff was instructed to report hypo‐ or hyperglycaemic events requiring the intervention of the medical staff.

This trial was not registered in any public domain because of a material error of one of the investigators. The protocol has been submitted and deposited in the database of the Florence Ethical Board since 27th October 2021, and it is available as Supporting Information S1.

The primary endpoint of this trial was the change from baseline to end of study in the percent time in range (TIR) between 70 and 180 mg/dL in 2 weeks, measured with CGM. Such outcome was chosen as it is a common CGM parameter used for the definition of good glycaemic control.^7^ Change from baseline in 2‐week TIR was compared between study arms. Data from CGM were considered valid if a minimum of 80% of glucose readings was available. FreeStyle Libre (and LibrePro, which is the same device in a blinded fashion) has a mean absolute relative difference [MARD] relative to self‐monitoring of blood glucose of 12.3% and 12.7%, respectively.^8^, ^9^

Secondary outcomes included the differences in the time spent above range (TAR) and below range (TBR), mean glucose levels (from CGM readings), glucose coefficient of variation (CV) and haemoglobin A1c (HbA1c). HbA1c was measured (with the Menarini high‐performance Liquid Chromatography Analyser) at the beginning and at the end of the study. In addition, the number of hypoglycaemic events was assessed (as reported by nurses of the nursing homes). The investigators decided to avoid the usual distinction of severe and non‐severe hypoglycaemia since the definition of severe hypoglycaemia (i.e., need of help from third parties) applies to a large majority of hypoglycaemic episodes in elderly people with multiple comorbidities and/or cognitive dysfunction, as many of the nursing home residents.

Baseline and clinical characteristics of enrolled individuals are presented as mean (standard deviation [SD]) or median (interquartile range) for continuous variables and count (percentage) for discrete variables and compared using Student unpaired *t* and Mann–Whitney tests, whenever appropriate, for continuous variables, and chi square tests for discrete variables. Non‐parametric Wilcoxon and Mann–Whitney tests were applied to compare glycaemic and CGM‐related endpoints in each study group and between the two study groups, respectively. A *p* value of less than 0.05 was considered significant for primary endpoint; applying Bonferroni correction for multiple comparisons for the reduction of type I errors, a *p* value <0.008 was considered statistically significant for secondary endpoints. All statistical analyses were performed using SPSS 29.0.

Sample size was calculated to be 84, to provide a power of at least 80% to detect a difference in mean TIR level between treatment groups, assuming a SD of 16.1,^9^ a two‐sided *α* level of 0.05, a 6% dropout rate and a change of 5% in TIR as clinically significant. Participant data were analysed according to their randomisation assignment (i.e., by intention to treat).

## 3 RESULTS

Eighty‐five patients from 16 nursing homes in Italy (Table S1 and Figure S2) were enrolled and randomised between January 2022 and September 2024. One of the subjects who had been randomised to the intervention group died 54 days after enrolment, because of sudden cardiac arrest (unrelated to study treatment); this patient was excluded from the efficacy analysis, which was therefore performed on 84 patients, 42 in the intervention and 42 in the control arm. No other patient was lost at follow‐up, and CGM data were complete for all enrolled participants. The trial was concluded when the pre‐defined sample size was reached. The study flow diagram is reported in Figure S3. No significant differences between groups were observed in the baseline characteristics, including age, sex, HbA1c, number of drugs prescribed for other concomitant diseases.

### 3.1 Principal endpoint

**FIGURE 1:**
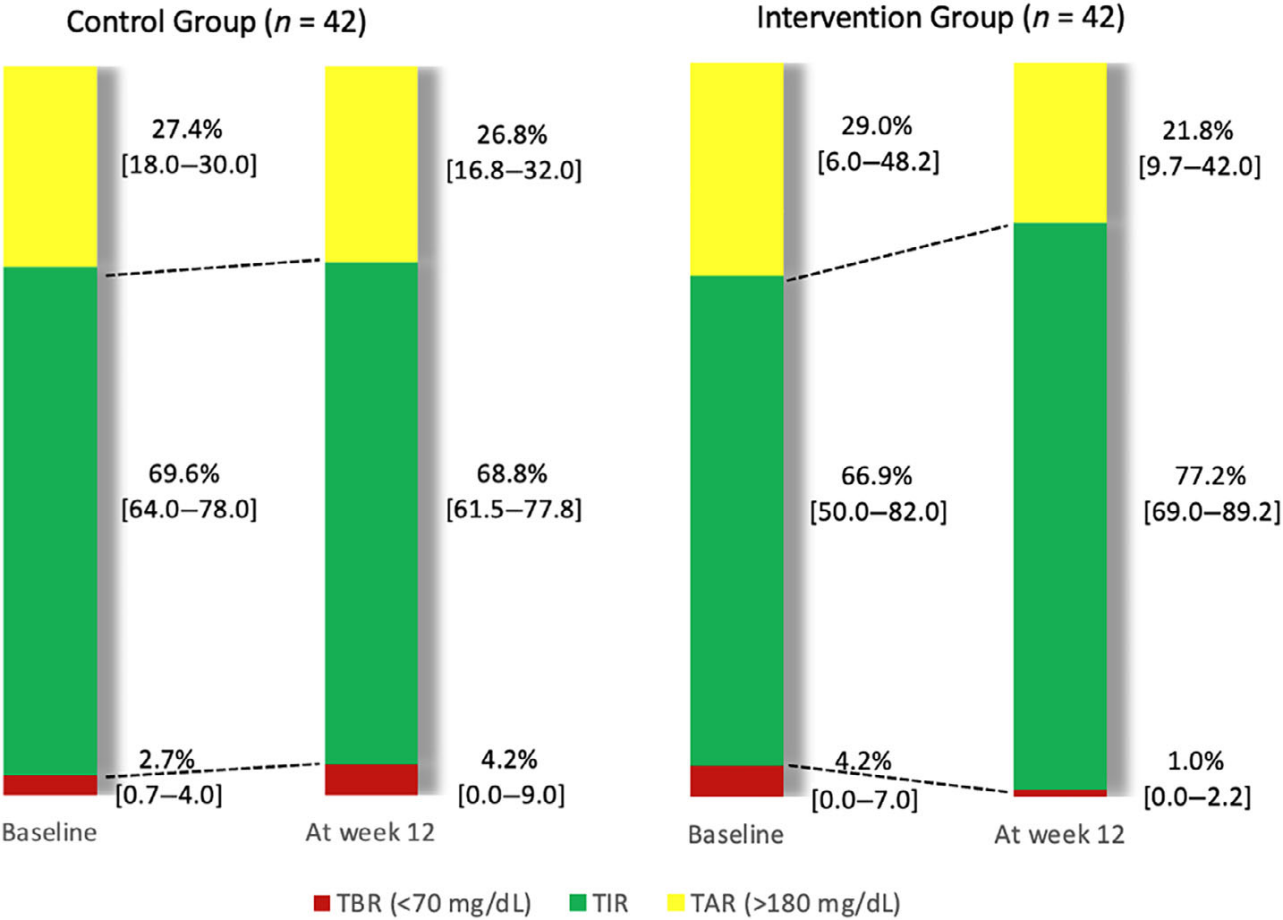
Continuous glucose monitoring data by study group. TAR, time above range; TBR, time below range; TIR, time in range.

Available sensor data were above the pre‐defined 80% threshold in both observation periods (beginning and end of study) in all patients enrolled, who could therefore be included in the analysis. TIR (% TIR) increased significantly in the intervention, but not in the control group (Figure 1). The difference between pre‐trial and the last 2 weeks of study was +10.3 [0; +16] and −0.8 [−8; +12] % in the intervention and control group, respectively (*p* = 0.010) (Table 1).

**TABLE 1:** Continuous glucose monitoring data, glucose control and mean daily insulin doses by study group.

	Control (*n* = 42)				Intervention (*n* = 42)				*p* between groups
	Pre	Post	Δ	*p*	Pre	Post	Δ	*p*	
TIR (%)	69.6 [64.0–78.0]	68.8 [61.5–77.8]	−0.8 [−8.0–12.0]	0.845	66.9 [50.0–82.0]	77.2 [69.0–89.2]	10.3 [0–16.0]	<0.001*	0.010*
TAR (%)	27.4 [18.0–30.0]	26.8 [16.8–32.0]	−0.5 [−14‐‐1.0]	0.357	29.0 [6.0–48.2]	21.8 [9.7–42.0]	−7.3 [−10‐‐1.1]	<0.012*	0.513
TBR (%)	2.7 [0.7–4.0]	4.2 [0.0–9.0]	+1.5 [1.0–6.1]	0.033*	4.2 [0.0–7.0]	1.0 [0.0–2.2]	−3.1 [−6.0–0.0]	<0.001*	0.007*
CV (%)	32.1 [27.8–36.0]	31.7 [27.3–34.6]	−0.3 [−3.6–3.9]	0.763	29.8 [24.3–35.5]	29.6 [25.3–32.6]	−0.2 [−5.5–4.1]	0.883	0.694
Mean glucose (mg/dL)	158 [148–167]	153 [121–166]	−5.1 [−34–2.0]	0.410	152 [114–179]	144 [120–156]	−8.4 [−17–7.0]	0.108	0.858
HbA1c (mmol/mol)	55.7 [52.0–61.0]	53.0 [47–56]	−2.7 [−13.0–0.0]	0.148	59.6 [49.7–56.0]	50.7 [44.2–55.5]	−9.0 [−21‐‐5.0]	<0.001*	0.028*
Mean daily insulin units (IU/day)	27.1 [18.0–32.5]	27.1 [18.0–34]	−0.0 [0.0–0.0]	0.881	39.2 [23.7–50.0]	35.2 [23.7–50.0]	−3.0 [−9.2–2.0]	0.002*	0.002*

*Note*: Data are expressed as median [quartile].

Abbreviations: CV, coefficient of variation; HbA1c, haemoglobin A1c; TAR, time above range (>180 mg/dL); TBR, time below range (<70 mg/dL); TIR, time in range (70–180 mg/dL).

* Statistically significant (for thresholds of signficance, see Methods).

These results are summarised in Figure 1.

### 3.2 Secondary efficacy endpoints

A significant reduction of TAR was observed in the intervention group, without significant differences between treatment arms. Conversely, TBR showed a reduction in the intervention arm (−3.1 [−6; 0] %) and an increase in the control arm (+1.5 [−1; +6%]), with a significant (*p* = 0.007) difference between groups (Figure 1). No significant effect of treatment was detected on mean glucose and glucose CV. HbA1c showed a significant reduction in the intervention group (−9 [−21; −5] mmol/mol), but not in the control group (−2.7 [−13; 0] mmol/mol), with a non‐significant (*p* = 0.028) between‐group difference. The intervention was also associated with a reduction of insulin doses (−3 [−9.2; −2] IU/day vs. control; *p* = 0.002) (Table 1). These results are summarised in Figure 1.

### 3.3 Safety endpoints

Only one serious adverse event was reported. This was a fatal event of myocardial infarction, in one 86‐years old participant with a history of prior myocardial infarction and coronary angioplasty, in the intervention group, that was considered unrelated to treatment allocation by the investigators; in the 2 h prior to the onset of symptoms, recorded glucose levels were in the 112–147 mg/dL range, excluding that the event could be related to glucose excursions. Two mild adverse events (both mild local inflammatory reactions at the site of sensor, one in the intervention and one in the control arm, which did not require the interruption of glucose monitoring) were also reported. In both cases, the sensor was removed and a new sensor was applied in a different site; no specific treatment was applied for the inflammatory reaction, which resolved spontaneously within 5 days. The intervention was associated with a reduction in the number of hypoglycaemic episodes, which was not observed in the control group; the difference between the pre‐trial and the last 2 weeks of the study was −1 [−4; 0] and 0 [0; +3] episodes in the intervention and control group, respectively (*p* = 0.082 for between‐group comparison).

## 4 DISCUSSION

This randomised clinical trial showed for the first time the efficacy and safety of a telemedicine‐based management of basal‐bolus insulin therapy in nursing home residents affected by T2D. Remote management of insulin treatment was associated with a clinically relevant improvement of glycaemic control as compared to SoC.

Most current guidelines recommend higher targets for glucose and HbA1c in older, fragile patients, particularly when insulin‐treated.^10^, ^11^, ^12^ In fact, the advantages of accurate glycaemic control in the prevention of complications are relatively smaller in subjects with a shorter life expectancy, whereas risks related to hypoglycaemia can be more relevant. Although non‐insulin drugs which are not associated with hypoglycaemia could be much easier to manage in elderly patients,^13^, ^14^ insulin treatment is often required to achieve sufficient glucose control in older patients with a long duration of diabetes,^3^ because of a progressive, age‐related decline in insulin secretion and/or because of concomitant contraindications to non‐insulin medications.^15^

Most patients enrolled in this trial showed acceptable levels of HbA1c and TIR. Despite this fact, the intervention determined a further improvement of those parameters. In addition, a relevant reduction of TBR and of the number of hypoglycaemic episodes was observed. In fact, prior to the intervention, many patients experienced repeated episodes of glucose below range, not always clinically detected as hypoglycaemia. Symptoms of hypoglycaemia can be blunted in patients with advanced age and/or long duration of diabetes,^16^ and cognitively impaired individuals can be incapable of communicating symptoms of hypoglycaemia. It can also be speculated that in some of the patients enrolled, daily insulin needs were reduced after admission to nursing homes because of changes in nutritional habits or comorbidities (e.g., because of weight loss related to cognitive decline), and that insulin doses had not been adjusted accordingly, leading to an increased risk of hypoglycaemia. The intervention appeared to be effective in correcting overtreatment with insulin, as suggested by the observed reduction of daily insulin doses; however, this result needs cautious interpretation, as the control group showed, by chance, a lower mean baseline insulin dose, generating the possibility that the reduction in the intervention group is an effect of regression to the mean. The reduction of time in hypoglycaemia is potentially very relevant, considering that low glucose is associated with falls^17^ and fractures.^18^ However, the present study was not appropriately sized for the assessment of such endpoints.

The role of specialists in the management of T2D is variable across countries and healthcare systems. In Italy, general practitioners are in charge of the management of T2D, but they periodically refer patients on basal‐bolus insulin therapy to specialist consultants for supervision and educational reinforcement. Patients with comorbidities that hamper access to outpatient clinics have a lower probability of being visited by specialists; this is the case of many nursing home residents. Telemedicine offers an option for reaching those individuals with specialist care. In fact, although telemedicine can be a useful support for the management of wider populations of subjects with T2D^19^ its relevance could be even greater in sub‐populations with greater obstacles to access specialist care.

The use of glucose sensors relieves personnel of the nursing home from the need of frequent measurements of capillary blood glucose, providing physicians with a relevant amount of data for an accurate adjustment of insulin doses. The greater availability of monitoring data could have been a key factor for the improvement of insulin dose adjustment and therefore of glucose control in the treatment arm. A recent trial failed to detect improvements of glucose control with CGM in hospitalised elderly patients,^20^ in apparent contrast with present data; however, in that study patients in the control arm received an accurate monitoring of blood glucose, as established by the protocol, and insulin doses were adjusted by the same physicians, using the same algorithms, in both treatment arms.

The use of CGM can improve glucose control, allowing for a timely recognition of hypoglycaemia and a prompt correction of hyperglycaemia.^5^ The design of the present trial does not allow a discrimination between the benefits of CGM per se and those of telemedicine. However, prior trials performed on patients with T2D in long‐term care facilities showed that remote adjustment of medication by specialists improves glucose control,^21^ whereas the use of CGM per se, when compared with traditional capillary blood glucose monitoring, does not modify glycaemic outcomes when insulin is adjusted by the same physicians.^20^ The role of telemedicine, facilitating access to specialists, seems therefore to prevail over the potential benefits on glucose control of CGM per se. In fact, although patients in the control group also had access to specialist care, free of charge, if deemed necessary by their physicians, it is likely that most of them did not exploit this possibility. Another point possibly affecting the interpretation of results is that the sensor used in the study, that is, a flash glucose monitoring device without alarms for hypo‐ or hyperglycaemia, is different from most of the presently available devices, which include alarms. It is possible that benefits would have been even greater with alarmed devices, but this hypothesis needs to be specifically investigated. Another limitation of the present study was that insulin dose adjustments in the treatment arm were remotely performed by specialists; it is conceivable that in other contexts, non‐specialists (i.e., general practitioners) would be in charge of such tasks. In addition, participating nursing homes were not representative of the whole area. However, the inclusion of several nursing homes in different parts of the region increases the potential applicability of results.

The main limitation of the study is the lack of a timely registration in a public repository, which allows for selective publication, introducing a bias. This limit, which was unintentional and determined by a material error of one of the investigators, represents a major issue, since the lack of publicity of ongoing trials allows for selective publication. In addition, despite randomisation, baseline insulin doses in the intervention arm were quantitatively higher than in the control arm, allowing more room for treatment optimisation.

Apart from costs for the acquisition of sensors, the resources needed for the investigational approach are limited. The workload for personnel of the nursing homes is not greater, and it may actually be smaller than that required for traditional capillary blood glucose measurements; the time required for physicians for adjustment of insulin doses is minimal. This approach seems therefore feasible and potentially cost‐effective. However, specific economic evaluations should be performed in different healthcare systems. In fact, the cost of devices is heterogeneous across different countries and healthcare providers, and personnel costs are also variable. A cost‐effectiveness assessment, which goes beyond the aims of the present study, should also include potential savings determined by the improvement of glucose control.

In conclusion, remote insulin dose adjustment based on interstitial glucose monitoring ameliorates glucose control in nursing home residents with T2D on basal‐bolus insulin therapy. Further, larger scale trials are needed to assess the potential benefits of this approach on other outcomes, such as falls.

## AUTHOR CONTRIBUTIONS

EM and ID conceptualised the study, researched and analysed the data and wrote the manuscript. RN and GDC participated to trial organization and contacts with nursing homes, contributed to data analysis and discussion of results, and reviewed the manuscript. The randomisation list was prepared and maintained by an investigator (GAS) who did not have any direct contact with enrolled patients. When a new patient was enrolled, the investigator in charge transmitted the initials to GAS. by email, receiving the allocation group in return by the same means. Patients in the intervention group performed continuous glucose monitoring (CGM) monitoring (Freestyle, Abbott) during the entire study period; adjustment of insulin doses was performed remotely by a specialist (ID, CDP, CM and GGDV), using information from CGM profiles, on a weekly basis. DS, CGP, VV, LD and FP instructed patients and healthcare personnel of the nursing homes for the management of CGM devices and of study procedures, and collected data on patients' records. All the authors reviewed the manuscript and edited the manuscript. EM is the guarantor of this work and, as such, had full access to all the data in the study and takes responsibility for the integrity of the data and the accuracy of the data analysis.

## FUNDING INFORMATION

This study has received research grant support for investigator‐initiated studies from Abbott Diabetes Care provided continuous glucose sensors and readers but had no role in the design of this study, its data collection, analysis or findings.

## CONFLICT OF INTEREST STATEMENT

The authors declare no conflicts of interest.

## Supporting information


**Data S1.** Supporting Information.

## Data Availability

The data are available on request from the authors.

## References

[1] Arnold SV , Lipska KJ , Wang J , Seman L , Mehta SN , Kosiborod M . Use of intensive glycemic management in older adults with diabetes mellitus. J Am Geriatr Soc. 2018;66(6):1190‐1194. doi:10.1111/jgs.15335 29633237 PMC7032960

[2] Idrees T , Castro‐Revoredo IA , Migdal AL , Moreno EM , Umpierrez GE . Update on the management of diabetes in long‐term care facilities. BMJ Open Diabetes Res Care. 2022;10(4):e002705. doi:10.1136/bmjdrc-2021-002705 PMC9305812 35858714

[3] Weiner JZ , Gopalan A , Mishra P , et al. Use and discontinuation of insulin treatment among adults aged 75 to 79 years with type 2 diabetes. JAMA Intern Med. 2019;179(12):1633‐1641. 31545376 10.1001/jamainternmed.2019.3759 PMC6763990

[4] Dicembrini I , Cosentino C , Monami M , Mannucci E , Pala L . Effects of real‐time continuous glucose monitoring in type 1 diabetes: a meta‐analysis of randomized controlled trials. Acta Diabetol. 2021;58(4):401‐410. 32789691 10.1007/s00592-020-01589-3

[5] Jancev M , Vissers TACM , Visseren FLJ , et al. Continuous glucose monitoring in adults with type 2 diabetes: a systematic review and meta‐analysis. Diabetologia. 2024;67(5):798‐810. 38363342 10.1007/s00125-024-06107-6 PMC10954850

[6] Moher D , Hopewell S , Schulz KF , et al. CONSORT 2010 explanation and elaboration: updated guidelines for reporting parallel group randomised trials. BMJ. 2010;340:c869. doi:10.1136/bmj.c869 20332511 PMC2844943

[7] Battelino T , Danne T , Bergenstal RM , et al. Clinical targets for continuous glucose monitoring data interpretation: recommendations from the international consensus on time in range. Diabetes Care. 2019;42(8):1593‐1603. 31177185 10.2337/dci19-0028 PMC6973648

[8] Staal OM , Hansen HMU , Christiansen SC , Fougner AL , Carlsen SM , Stavdahl Ø . Differences between flash glucose monitor and fingerprick measurements. Biosensors. 2018;8(4):93. 30336581 10.3390/bios8040093 PMC6316667

[9] Nakagawa Y , Hirota Y , Yamamoto A , et al. Accuracy of a professional continuous glucose monitoring device in individuals with type 2 diabetes mellitus. Kobe J Med Sci. 2022;68(1):E5‐E10. 36647081 PMC10117624

[10] Munshi MN , Florez H , Huang ES , et al. Management of diabetes in long‐term care and skilled nursing facilities: a position statement of the American Diabetes Association. Diabetes Care. 2016;39(2):308‐318. 26798150 10.2337/dc15-2512 PMC5317234

[11] Sinclair A , Morley JE , Rodriguez‐Mañas L , et al. Diabetes mellitus in older people: position statement on behalf of the International Association of Gerontology and Geriatrics (IAGG), the European Diabetes Working Party for Older People (EDWPOP), and the International Task Force of Experts in Diabetes. J Am Med Dir Assoc. 2012;13(6):497‐502. 22748719 10.1016/j.jamda.2012.04.012

[12] Mannucci E , Candido R , Monache LD , et al. 2023 update on Italian guidelines for the treatment of type 2 diabetes. Acta Diabetol. 2023;60(8):1119‐1151. 37233852 10.1007/s00592-023-02107-x PMC10290044

[13] Umpierrez GE , Cardona S , Chachkhiani D , et al. A randomized controlled study comparing a DPP4 inhibitor (linagliptin) and basal insulin (glargine) in patients with type 2 diabetes in long‐term care and skilled nursing facilities: linagliptin‐LTC trial. J Am Med Dir Assoc. 2018;19(5):399‐404.e3. doi:10.1016/j.jamda.2017.11.002 29289540 PMC6093296

[14] Pasquel FJ , Powell W , Peng L , et al. A randomized controlled trial comparing treatment with oral agents and basal insulin in elderly patients with type 2 diabetes in long‐term care facilities. BMJ Open Diabetes Res Care. 2015;3(1):e000104. 10.1136/bmjdrc-2015-000104 PMC4553905 26336609

[15] Fonseca VA . Defining and characterizing the progression of type 2 diabetes. Diabetes Care. 2009;32(suppl 2):S151‐S156. 19875543 10.2337/dc09-S301 PMC2811457

[16] Bremer JP , Jauch‐Chara K , Hallschmid M , Schmid S , Schultes B . Hypoglycemia unawareness in older compared with middle‐aged patients with type 2 diabetes. Diabetes Care. 2009;32(8):1513‐1517. 19487634 10.2337/dc09-0114 PMC2713637

[17] Lee AK , Juraschek SP , Windham BG , et al. Severe hypoglycemia and risk of falls in type 2 diabetes: the atherosclerosis risk in communities (ARIC) study. Diabetes Care. 2020;43(9):2060‐2065. 32611607 10.2337/dc20-0316 PMC7440903

[18] Hung YC , Lin CC , Chen HJ , et al. Severe hypoglycemia and hip fracture in patients with type 2 diabetes: a nationwide population‐based cohort study. Osteoporos Int. 2017;28(7):2053‐2060. 28374044 10.1007/s00198-017-4021-4

[19] Hangaard S , Laursen SH , Andersen JD , et al. The effectiveness of telemedicine solutions for the management of type 2 diabetes: a systematic review, meta‐analysis, and meta‐regression. J Diabetes Sci Technol. 2023;17(3):794‐825. 34957864 10.1177/19322968211064633 PMC10210100

[20] Idrees T , Castro‐Revoredo IA , Oh HD , et al. Continuous glucose monitoring‐guided insulin administration in long‐term care facilities: a randomized clinical trial. J Am Med Dir Assoc. 2024;25(5):884‐888. 38460943 10.1016/j.jamda.2024.01.031 PMC11283256

[21] Gerber BS , Biggers A , Tilton JJ , et al. Mobile health intervention in patients with type 2 diabetes: a randomized clinical trial. JAMA Netw Open. 2023;6(9):e2333629. 37773498 10.1001/jamanetworkopen.2023.33629 PMC10543137

