# Supplementary material for: Efficacy of telemedicine on glycaemic control in nursing home residents with type 2 diabetes on basal‐bolus insulin therapy: A randomised controlled trial
Source: Diabetes Obes Metab. 2026 Jan 26;28(4):3202–8. doi: 10.1111/dom.70511 (PMC12992162; doi:10.1111/dom.70511)

|  | **All n=84** | **Intervention n=42** | **Control n=42** | **Pearson R/Student’s T** | **P** |
| --- | --- | --- | --- | --- | --- |
| **Age** *(years – mean ± SD)* | 80.5 ± 11.8 | 81.8 ± 12.9 | 78.4 ± 9.8 | -1.39 | 0.17 |
| **Sex**:   - **Male** *[n, (%)]* - **Female** *[n, (%)]* | 26 (30.9)  58 (69.1) | 15 (35.7)  27 (64.3) | 11 (26.2)  31 (73.8) | 0.89 | 0.15 |
| **Number of concomitant drugs:**   - **≧ 5** *[n, (%)]* - **< 5** *[n, (%)]* | 81 (96.4)  3 (3.6) | 40 (95)  2 (5) | 41 (97.6)  1 (2.4) | 0.35 | 0.56 |
| **Cardiovascular diseases*** *[n, (%)]* | 78 (92.9) | 40 (95.2) | 38 (90.5) | 0.72 | 0.40 |
| **HaA1c** (mmol/mol- *mean ± SD*) | 58.34±11.6 | 59.6±13.3 | 56.7±9.0 | -1.1 | 0.28 |
| **Central nervous system diseases** *[n, (%)]* | 55 (65.5) | 30 (71.4) | 25 (59.5) | 1.31 | 0.25 |
| **Neoplasia** *[n, (%)]* | 11 (13.1) | 8 (19) | 3 (7.1) | 2.6 | 0.11 |
| **Fracture-prone osteoporosis** *[n, (%)]* | 10 (11.9) | 4 (9.5) | 6 (14.3) | 0.45 | 0.74 |
| **COPD** *[n, (%)]*   - With Oxigen-therapy *[n, (%)]* | 15 (17.9)  12 (14.3) | 7 (16.7)  7 (16.7) | 8 (19.0)  5 (11.9) | 0.08  0.39 | 0.78  0.53 |
| **CKD on dialysis treatment** *[n, (%)]* | 3 (3.6) | 1(2.4) | 2 (4.8) | 0.35 | 0.56 |
| **Insulin dose at baseline** (IU) | 38.72±21.4 | 39.4±21.9 | 29.3±9.3 | 0.79 | 0.14 |
| **Basal Insulin analogues:**   - *1^st^ generation basal analogue* *[n, (%)]* - *2^nd^ generation basal analogue [n, (%)]* | 22(26)  62(74) | 12 (29)  30 (71) | 10 (24)  32 (76) | 1.02  0.21 | 0.31  0.65 |
| **Diabetes duration:**   - < 5 years *[n, (%)]* - 5 - 10 years *[n, (%)]* - > 10 years *[n, (%)]* | 5 (6.0)  26 (30.9)  53 (63.1) | 3 (7.1)  12 (28.6)  27 (64.3) | 2 (4.8)  14 (33.3)  26 (61.9) | 0.21  0.22  0.05 | 0.65  0.64  0.82 |

**Supplementary Material.**

**Table S1:** Baseline clinical characteristics in each study arm. Cardiovascular diseases: hypertension, ischemic heart disease, ischemic encephalopathy, heart failure. Central nervous system diseases: cognitive decline, psychiatric disorders. COPD: chronic obstructive pulmonary disease; CKD: chronic kidney disease; 1st generation basal analogue: determir or U-100 glargine; 2nd generation basal analogue: degludec or U-300 glargine.

Supplementary Material. Fig. 1. Consort 2010 Checklist.


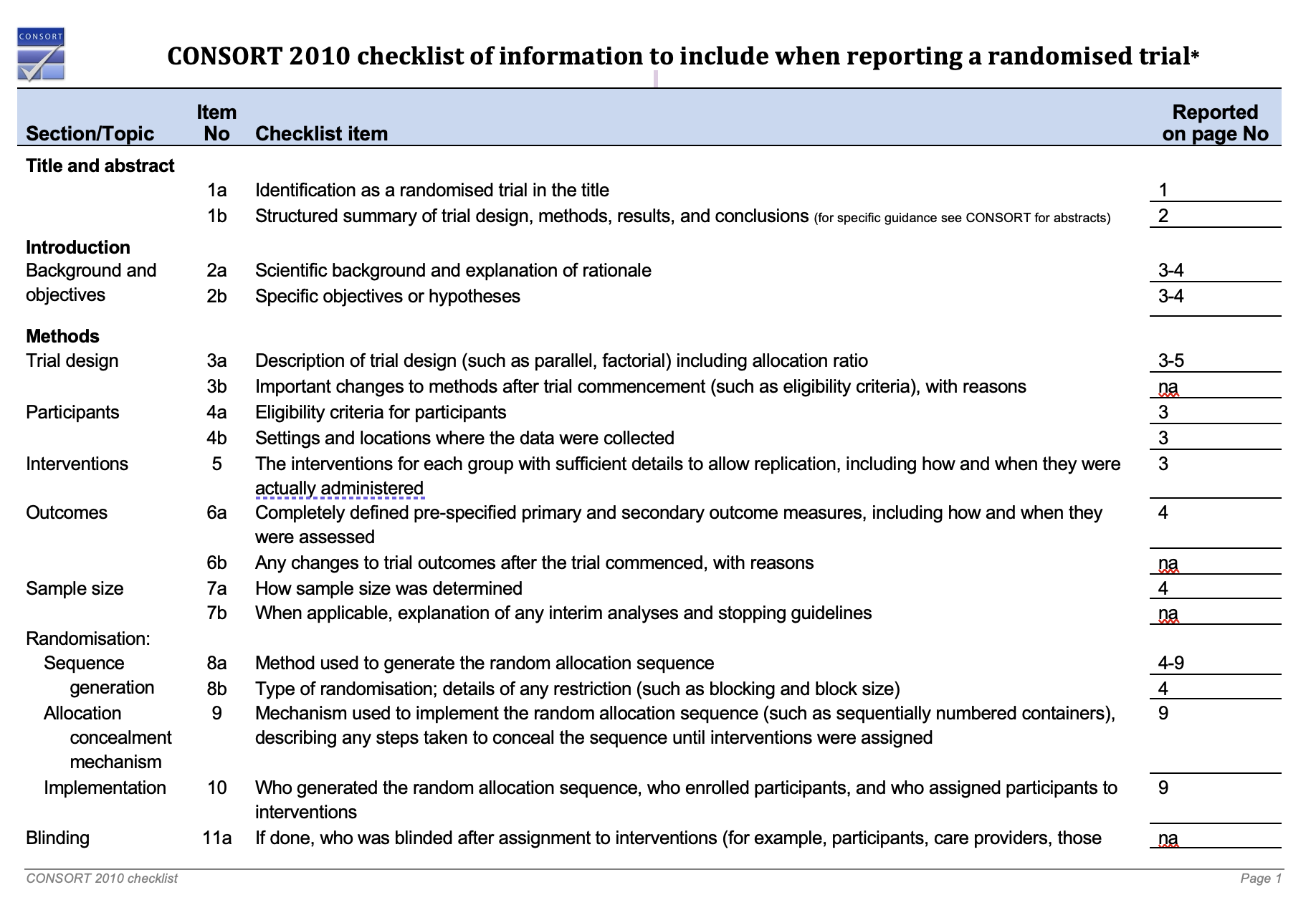


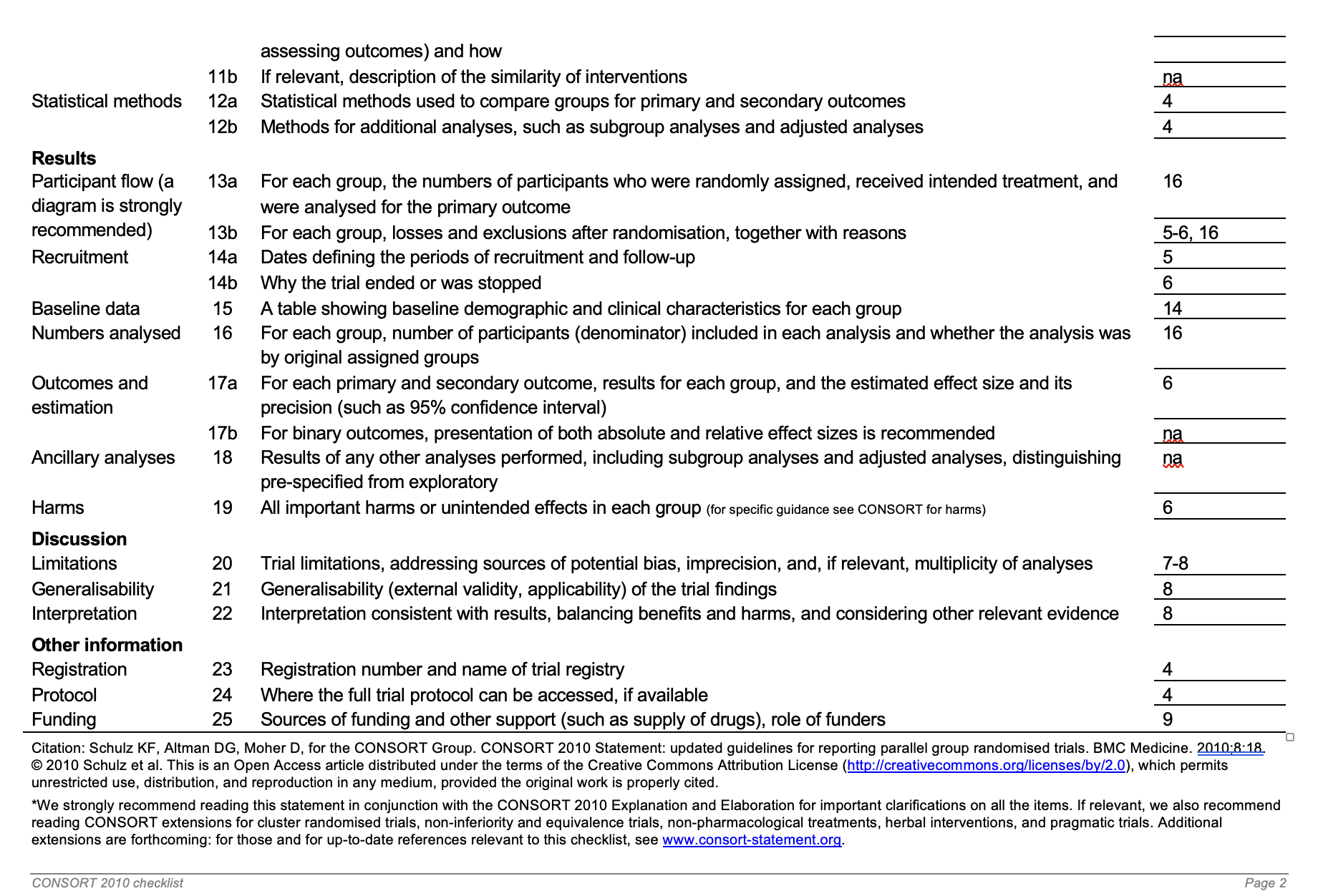


Supplementary Material. Fig. 2. List of Nursing homes.


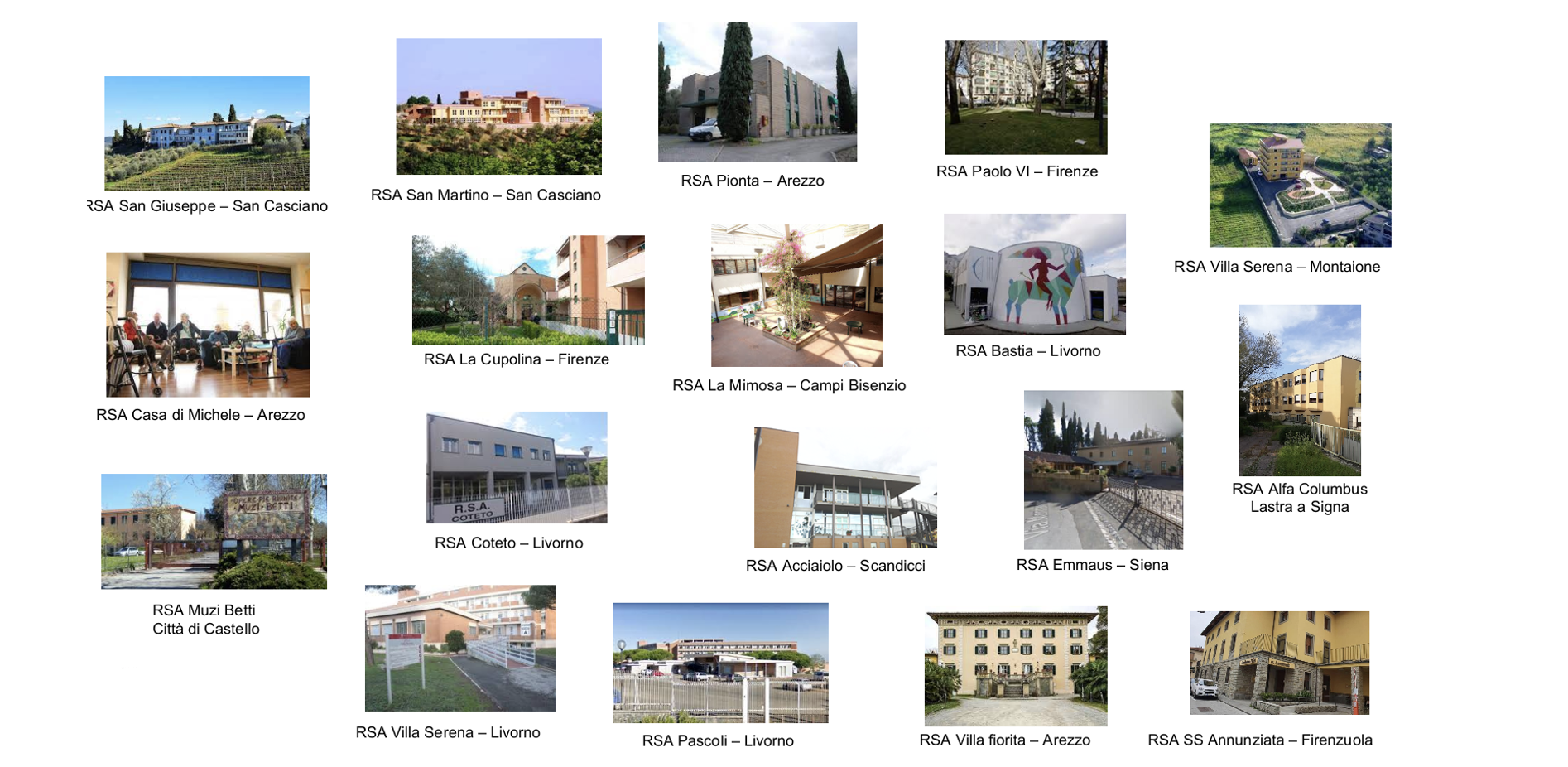


Supplementary Material. Fig. 3. Study Flow Diagram.


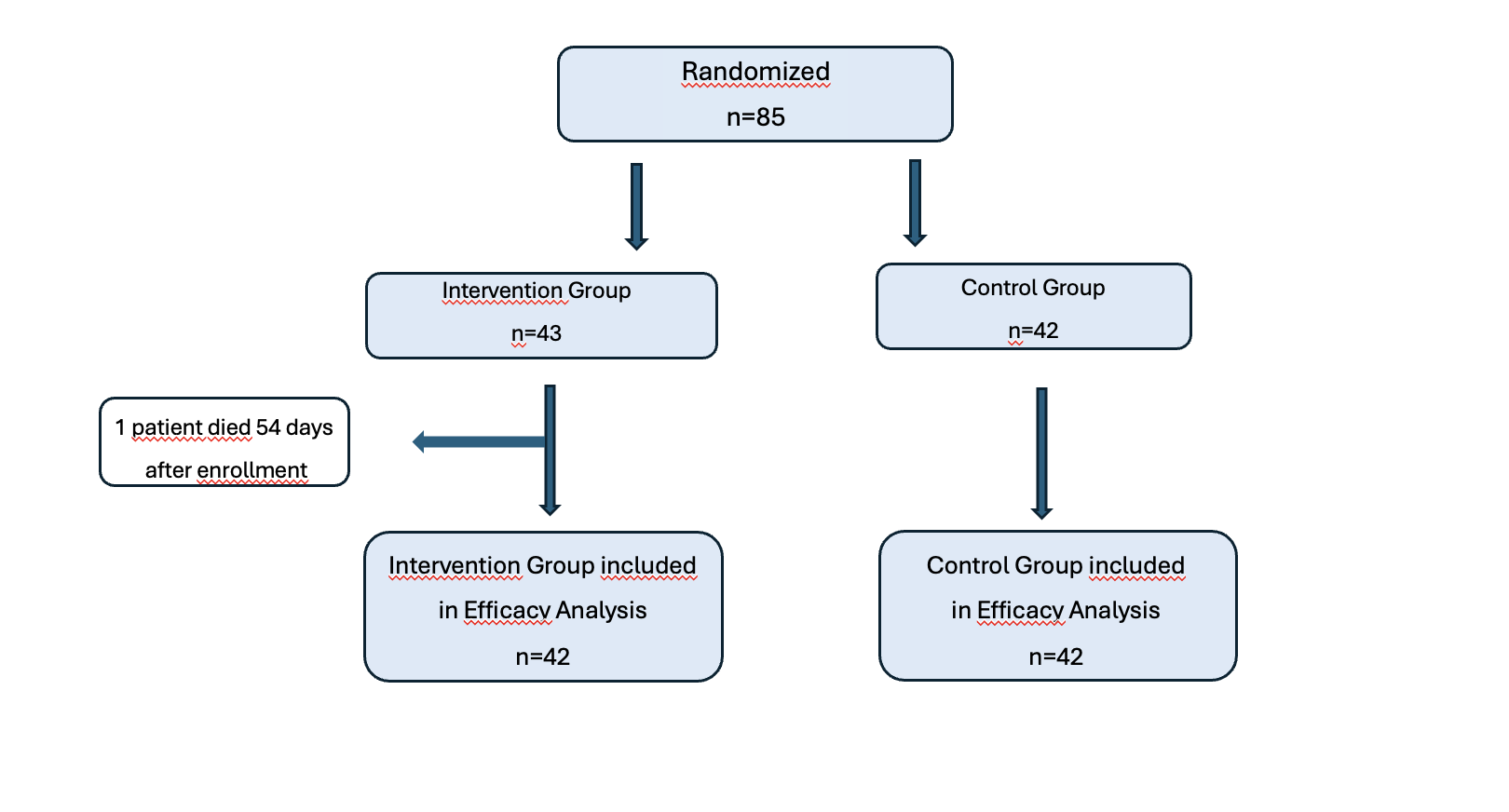

Supplement: Supplementary file 1 — Data S1. Supporting Information. [file DOM-28-3202-s001.docx]
